# Intensity Modulated Radiotherapy (IMRT) in the postoperative treatment of an adenocarcinoma of the endometrium complicated by a pelvic kidney

**DOI:** 10.1186/1748-717X-1-44

**Published:** 2006-11-20

**Authors:** Marcus S Castilho, Alexandre A Jacinto, Gustavo A Viani, Andre Campana, Juliana Carvalho, Robson Ferrigno, Paulo ERS Novaes, Ricardo C Fogaroli, Joao V Salvajoli

**Affiliations:** 1 Department of Radiation Oncology, Hospital do Câncer A C Camargo, São Paulo, Brazil

## Abstract

**Background:**

Pelvic Radiotherapy (RT) as a postoperative treatment for endometrial cancer improves local regional control. Brachytherapy also improves vaginal control. Both treatments imply significant side effects that a fine RT technique can help avoiding. Intensity Modulated RT (IMRT) enables the treatment of the target volume while protecting normal tissue. It therefore reduces the incidence and severity of side effects.

**Case:**

We report on a 50 year-old patient with a serous-papiliferous adenocarcinoma of the uterus who was submitted to surgical treatment without lymph node sampling followed by Brachytherapy, and Chemotherapy. The patient had a pelvic kidney, and was therefore treated with IMRT.

So far, the patient has been free from relapse and with normal kidney function.

**Conclusion:**

IMRT is a valid technique to prevent the kidney from radiation damage.

## Background

Randomized trials have shown that Pelvic Radiotherapy (RT) as a postoperative treatment for intermediate and high risk endometrial cancer improves local regional control. Its impact on overall survival is still unknown. Intra-cavitary Brachytherapy also improves vaginal control. Both treatments, however, imply significant side effects that a fine technique can help avoiding. Intensity Modulated RT (IMRT) is the most efficient external beam RT delivery technique nowadays. Using a high gradient of radiation dose enables the treatment of the target volume while protecting normal tissues in an attempt to reduce the incidence and severity of side effects.

### Patient history

A 50-year old Caucasian woman was referred to the Radiation Oncology Department of Hospital do Cancer A C Camargo, São Paulo, Brazil, with Endometrial Cancer. Due to bilateral ovary mass she was submitted to exploratory laparotomy. During the surgical procedure, Total Abdominal Hysterectomy and Bilateral Salpingectomy and Oophorectomy (TAH/BSO) were performed. The pathological analysis revealed a mucinous cystic adenoma in her left ovary and an endometrioid cyst in her right ovary (no evidence of malignancy). The endometrium presented a solid, *Serous *Papiliferous Adenocarcinoma, poorly differentiated, compromising the inner half of the myometrium with extension to the upper endocervix. There was no lymph vascular space invasion and the margins were not compromised.

She was classified as IIA by FIGO criteria [1] and received 6 cycles of Carboplatin and Paclitaxel, followed by 29 Gy of High Dose Rate Brachytherapy (HDR BT) prescribed on the vaginal surface, divided in 4 fractions, with median dose to the rectum and bladder reference points of respectively 48 and 58%.

She was referred to our Institution because she had a Congenital Pelvic Kidney.

Static and dynamic Scintigrafic renal function studies were performed. They showed that the pelvic kidney was functioning perfectly – it absorbed 45% of the injected radioactive isotope.

A study plan for IMRT was led. It showed the dose to normal tissue and kidney was kept under tolerable limits. The patient was informed of the risks and benefits of proceeding with the treatment. The prescribed dose to cover 95% of the target volume (whole pelvic drainage and vaginal vault) was 45 Gy at 1.8 Gy per fraction.

Seven co-planar fields were chosen at an interval rotation of 50 degrees. Dynamic Multileaf Collimation was used. The target volume excluded the entire pelvic kidney and covered pelvic lymphatics from L5 down.

**Figure 1:**
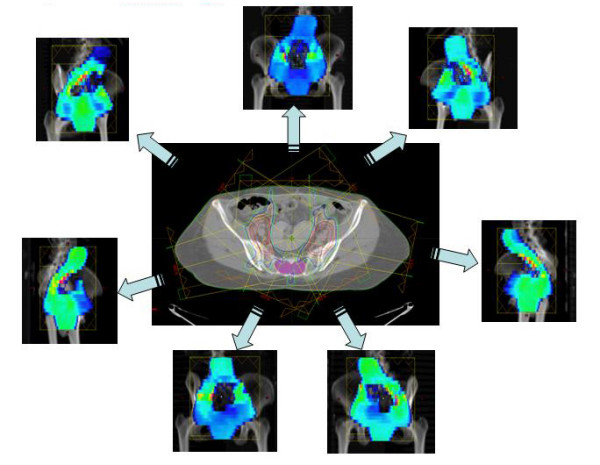
**Radiation fluence**. shows radiation fields, their fluence maps, and the resulting dose distribution on a section plane that includes the pelvic kidney.

**Figure 2:**
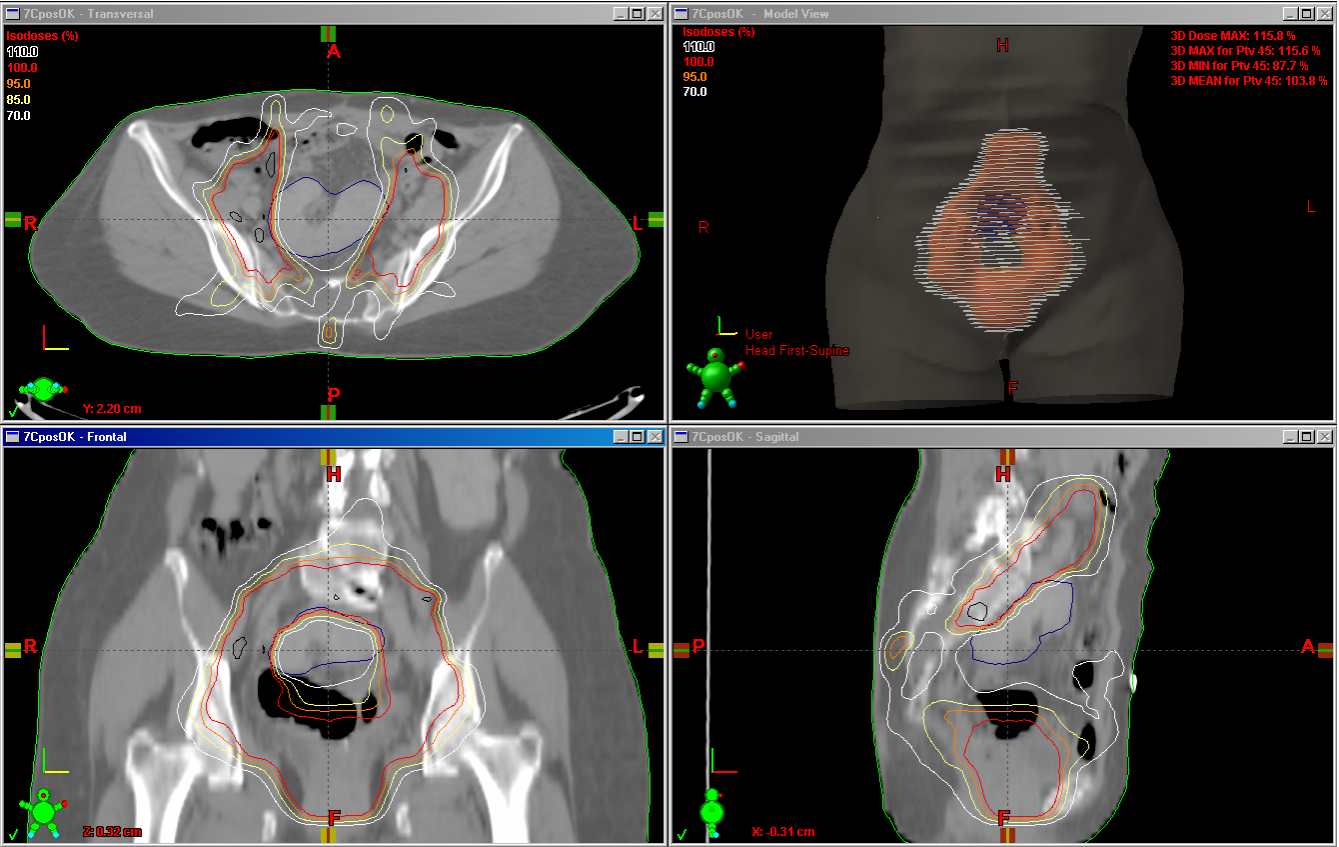
**Dose distribution**. shows the dose distribution for the 45 Gy prescribed dose.

**Figure 3:**
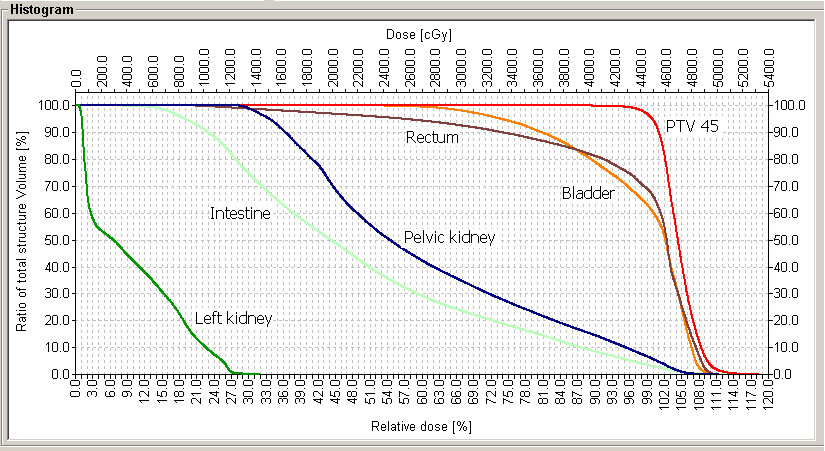
**Dose Volume Histogram**. shows the graphic of Dose Volume Histogram. The curves show the distribution for the PTV, rectum, bladder, intestines, pelvic kidney and left topic kidney.

RT field fluency is presented in figure 1, and Dose Distribution is presented in figure 2. The dose volume analysis (DVH) is presented in figure 3.

Planned dose distribution was verified dosimetrically and matched the software's calculation. The qualitative analysis of isodose curves was satisfactory too.

During treatment, the patient presented peri-anal radiodermitis (RTOG grade 1), increased bowel movements (up to 3 times/day), and a lowering in platelet count levels (75,000/mm3) which led to a 7 day treatment interruption at 37.8 Gy. She subsequently recovered with a platelet rise to 90,000/mm3 and the treatment was resumed. The renal function panel was unaltered during the whole RT course.

When last seen – 18 months after the end of RT – the patient was free from disease. She had normal kidney function, both by serum panel and isotopic nephrogram evaluation. The nephrogram did not show any changes compared to the initial exam.

## Discussion

Patients with a pelvic kidney should not receive RT unless it is a mainstream in the treatment of that type of tumor.

There are very few reports on treating pelvic kidneys patients with EBRT [2-8].

It is important to establish the need, and the benefits of RT to any patient with in such a condition.

### Pelvic kidney function

We used renal blood tests and isotopic nephrogram to access the patient's renal function. The scintigrafic study used static and dynamic assessment of glomerular and tubular function. Her right pelvic kidney took 45% of the radio labeled marker (DMSA/DTPA), and had normal excretion of it. Eighteen months after the treatment the kidney' uptake was unchanged.

Scintigraphic renograms have correlated with biochemical and clearance end points [9], and are adequate for this situation, as the other kidney is functioning well, and any effect on the pelvic kidney would be better seen with functional images rather than with functional biochemical exams.

### Benefit of adjuvant radiation and chemotherapy

The standard surgical treatment for uterine neoplasia consists of Radical Hysterectomy, bilateral salpingo oophorectomy, and lymphadenectomy or lymph node sampling.

In this case, the surgical approach was not radical in intent because the uterine neoplasia was an incidental finding. Therefore, the lymph node status was not known. In this setting, the benefit of re-operation is unclear and not evidence-based. The prospective PORTEC trial [10] has directly tested the benefit of RT for patients without lymph node information. Patients with endometrial adenocarcinoma were randomized to receive postoperative pelvic EBRT, or no adjuvant therapy. They noticed a significant advantage in pelvic control for the adjuvant treatment arm with risk features (deep myometrial invasion, cervical canal extension, high grade histology, or lymph vascular space invasion), though not translated into survival benefit. The majority of failures occurred at the vaginal vault. This study did not evaluate specifically serous papiliferous tumors, but this subset of tumors is known to have a worse prognosis. This patient is classified as having a high risk tumor. It is considered a non- endometrioid tumor, not responsive to estrogenic castration. Metha and cols [11] have studied a group of women with stage I-II serous papiliferous tumors treated with surgery followed or not by adjuvant therapy. Though no variables were statistically correlated to prognosis, out of 13 women who did not receive RT/BT, 5 recurred in the pelvis (4 in the vagina, 1 in the lateral pelvis). In contrast, none of the patients who received RT/BT (total of 10) recurred in the pelvis. The 5-year pelvic recurrence free survival was 100 vs. 57%, with a p = 0.06.

This information and other published results suggesting a benefit of carboplatin/paclitaxel based chemotherapy for this histological type and the fact that this histological type of tumor carries a high risk of recurrence makes us believe that our patient did benefit from the adjuvant chemo-radiotherapy, including vaginal vault BT.

### Expected risks, side effects, and tolerance

Kidney tolerance to radiation dose highly depends on the irradiated volume.

Tolerance dose for a 5% chance of late adverse effect at 5 years is estimated to be 50 Gy for one third of the kidney, 30 Gy for two thirds, and 23 Gy for the whole kidney [12]. It increases to 50% late toxicity if two thirds are irradiated to a dose of 40 Gy or one third to a dose of 28 Gy.

As noted on the DVH (figure 3) these parameters have been respected in the present case.

The literature does not define the optimal treatment for patients with pelvic kidneys who need to undergo pelvic RT. We could find 7 case reports concerning this subject [2,4-8]. In 5 cases the primary tumor being treated was a uterine cervix carcinoma [2,6-8]. In 3 of them, the kidney was transplanted outside the pelvis, away from the RT target volume [2,7,8]. However, there was significant morbidity related to the procedure, especially regarding the graft vasculature, and the urinary tract. In one case an adenocarcinoma of the uterine cervix in a transplanted patient was treated initially with Intracavitary BT (low dose rate) followed by a modified field pelvic RT protecting the kidney, but partially compromising the RT target volume [6]. This patient relapsed on the border of the RT field.

Other reports of auto-transplantation followed by RT for inguinal-pelvic irradiation in a vulvar cancer patient, and for adjuvant treatment of a stage III operated rectal adenocarcinoma exists [4,5].

Although the preferred approach has not been established, no report exists on the use of high technology RT in an attempt to accomplish an adequate plan without moving the kidney out of the RT field. Conformal 3D RT has been developed to precisely study the combination of RT fields, and properly match the dose distribution to the CT visible tumor, while evaluating dose received by normal tissue, therefore predicting treatment tolerance. It is however limited in achieving these goals when the tumor is surrounded by normal tissues with low radiation resistance, or when the normal organ is in the middle of the RT port. In this setting IMRT has been shown effective, and its use for head and neck, thoracic, and abdominal treatments have been increasing.

We showed that IMRT is also a good alternative in such a complex situation. It has prevented the patient from undergoing an auto-transplantation procedure.

During treatment, this patient presented mild (common toxicity criteria grade 1) platelet complication. Lately there has been an increase in the use of IMRT to spare the blood marrow, providing that, in case of a relapse and need for new chemotherapy regimens, maintaining as much functioning marrow as possible presents another advantage of using IMRT. Roeske and cols have shown the main location of blood elements production in the pelvis [13], and it is possible to define these points as dose restriction points for the IMRT planning.

To our knowledge, this is the first report on the use of IMRT to spare a pelvic kidney without compromising a pelvic RT plan.

IMRT was a valid radiation technique to keep the pelvic kidney dose under acceptable dose volume constraints without compromising the target volume.

IMRT should be considered an option for treating pelvic fields in patients who present a pelvic kidney.
